# Free-living and particle-attached bacterial community composition, assembly processes and determinants across spatiotemporal scales in a macrotidal temperate estuary

**DOI:** 10.1038/s41598-022-18274-w

**Published:** 2022-08-16

**Authors:** Marion Urvoy, Michèle Gourmelon, Joëlle Serghine, Emilie Rabiller, Stéphane L’Helguen, Claire Labry

**Affiliations:** 1 Ifremer, DYNECO, 29280 Plouzané, France; 2 CNRS, IRD, Ifremer, UMR 6539, Laboratoire des Sciences de l’Environnement Marin (LEMAR), Université de Bretagne Occidentale, 29280 Plouzané, France

**Keywords:** Environmental sciences, Microbial ecology, Molecular ecology

## Abstract

Bacteria play an important role in biogeochemical cycles as they transform and remineralize organic matter. Particles are notable hotspots of activity, hosting particle-attached (PA) communities that can differ largely from their free-living (FL) counterparts. However, long-standing questions remain concerning bacterial community assembly processes and driving factors. This study investigated the FL and PA community compositions and determinants within the Aulne estuary and the Bay of Brest coastal waters (France). Our results revealed that the FL and PA community compositions greatly varied with salinity and season, explaining a larger part of the variance than the sampling fraction. Both the FL and PA communities were driven by deterministic assembly processes and impacted by similar factors. The FL-PA dissimilarity varied across space and time. It decreased in the estuarine stations compared to the freshwater and marine ends, and in summer. Interestingly, a significant proportion of the FL and PA communities' β-diversity and dissimilarity was explained by cohesion, measuring the degree of taxa co-occurrence. This suggested the importance of co-occurrence patterns in shaping the FL and PA community compositions. Our results shed light on the factors influencing estuarine bacterial communities and provide a first step toward understanding their biogeochemical impacts.

## Introduction

Bacterial communities are of critical importance in the ocean. Through their transformation of dissolved (DOM) and particulate organic matter (POM), they carry out functions that profoundly impact global biogeochemical cycles^1^. Consequently, one of the long-standing questions in microbial biogeography is how bacterial communities are shaped across spatiotemporal scales^2,3^. Bacterial communities are usually separated into free-living (FL) or particle-attached (PA) bacteria via size-filtration, although there are dynamic exchanges between the two communities^4^. Particles are considered hotspots of bacterial activities^5,6^. Indeed, PA communities are usually more active^6–9^ and have more diverse metabolic capacities^10–12^ than FL bacteria. These differences in functional capacities can be paralleled by differences in bacterial community composition (BCC): several studies have showed that PA bacteria are more diverse and taxonomically different from their FL counterparts^8,13,14^. However, other studies have reported that PA communities are not significantly different from FL bacteria^15–17^. Since few studies have addressed the seasonal variations in PA and FL communities, the factors driving their dissimilarities are not well characterized.

Bacterial community assembly is influenced by both deterministic and stochastic processes. Deterministic processes correspond to the ecological selection of species, driven by biotic (i.e., interactions among microorganisms) and abiotic (e.g., temperature) factors. Deterministic processes include homogeneous and heterogeneous selection that lead to more similar communities than what would be expected by chance, or less similar communities, respectively^18^. Stochastic processes are random events and include ecological drift, homogenizing dispersal, and dispersal limitation^3,18^. The BCCs and functions of PA and FL communities may be affected by stochastic and deterministic processes in different proportions, which has not been well characterized in marine ecosystems, especially across spatiotemporal scales^19–21^.

Several studies have matched patterns in BCC to abiotic factors such as temperature, salinity, organic matter quantity and composition or nutrient concentrations^16,19,22,23^. In addition, the quality and quantity of particles and nutrient concentrations have been suggested to drive FL-PA community dissimilarities^15,17,24,25^. However, despite their increasingly recognized importance, few studies have investigated the impact of biotic interactions among bacterial communities^26–28^. Due to their inner complexity, these interactions must be statistically inferred by correlating species' relative abundance^28^. To this end, different tools have been developed, such as co-occurrence networks^29,30^ or, more recently, cohesion metrics^28^. Positive and negative cohesion respectively reflect the degree of positive and negative co-occurrence within a community, thus providing insights into taxa associations^28^.

Estuaries are interesting zones to examine BCC and FL-PA dissimilarities. These highly dynamic ecosystems exhibit strong gradients that shape biological processes, and their high particle loads favor the establishment of highly productive PA bacterial communities^31,32^ that can constitute up to 90% of total bacterial cells^5^. In contrast, PA bacteria usually represent less than 5% of the bacterial community in most pelagic environments such as the open ocean^5^. Estuaries contain a complex mix of refractory terrestrial organic matter and labile autochthonous organic matter that is heavily transformed by both microbial and physicochemical processes^33,34^. Estuaries are thus vital components of the land–sea continuum, modulating the quality and quantity of organic matter exported to coastal areas^35^. Understanding BCC dynamics and their determinants along estuarine salinity gradients could help identify the microbial transformations of organic matter in these area, as well as their impacts on the adjacent coastal waters.

In this study, the FL and PA BCCs were investigated within the Aulne estuary (Brittany, France), which presents high bacterial production and hydrolytic activity levels resulting from particle-attached bacterial communities^31^. The objectives were to determine (i) the spatiotemporal variation in the FL and PA BCCs within the Aulne estuary; (ii) the assembly processes (stochastic vs. deterministic) driving the structuring of FL and PA communities along the salinity gradient; and (iii) the factors (biotic and abiotic) driving the FL and PA BCCs, as well as the FL-PA dissimilarity. To answer these questions, we examined the FL and PA BCCs in the Aulne estuary using 16S rRNA gene metabarcoding sequencing from fresh to marine waters, covering contrasting seasons. Our initial hypotheses were that i) BCC would be largely driven by salinity and ii) FL-PA dissimilarity would be controlled by POM quality.

## Materials and methods

### Study site

The Aulne River (northwest France) is 130 km long, drains an area of approximately 1800 km^2^ and supplies 63% of the freshwater inputs to the semi-enclosed Bay of Brest^36^. Its catchment comprises agricultural areas and meadows. The river flow regime ranges from 1 to more than 250 m^3^ s^−1^ (with a mean of 28 m^3^ s^−1^) and is constrained by a temperate oceanic climate, generating markedly higher precipitation in winter^37^. The macrotidal Aulne estuary ranges 30 km from the Guily-Glaz Dam to the river’s mouth. The important semidiurnal tidal amplitude (1.2–7.5 m) results in intense water depth variations, affecting particulate matter resuspension and deposition. The water residence time varies between 3 and 30 days^38^.

### Sampling strategy

**Figure 1:**
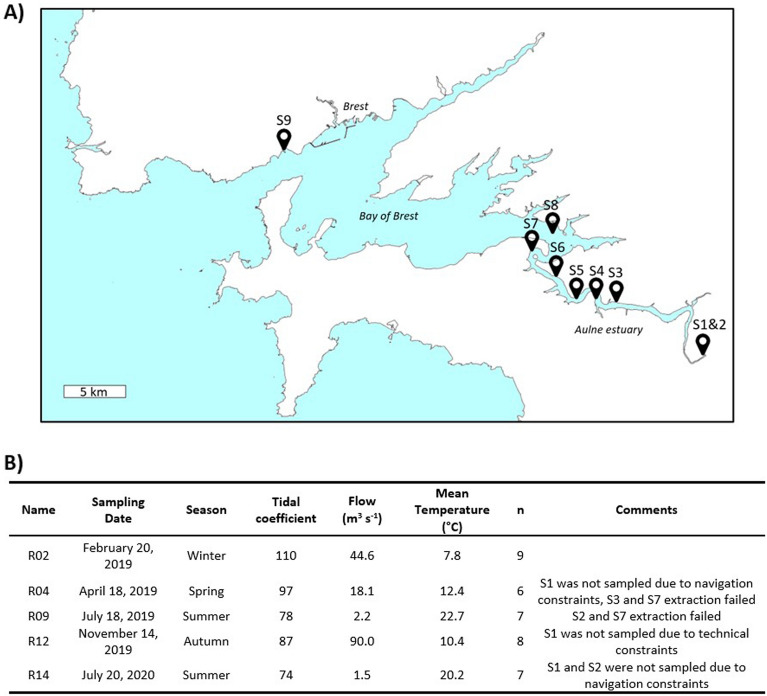
**(A)** Map of the Bay of Brest and the Aulne estuary; sampling dates and their associated tidal coefficient, river flow and mean water temperature. Freshwater (S1, S2) and marine water (S9) stations were collected at fixed locations, while the other stations were variable depending on the salinity. The locations of the April sampling (R04) stations are indicated as an example. Modified from data.shom.fr. (**B**) Sampling dates and their associated tidal coefficient, river flow and mean water temperature. n: number of sampled stations. n: number of sampled stations. Flow data were retrieved from http://www.hydro.eaufrance.fr/ at the Châteaulin station (J3821820).

The samplings were carried out at spring tide on February 20 (R02), April 18 (R04), July 18 (R09) and November 14 (R12), 2019, as well as on July 20, 2020 (R14), in the context of a monthly monitoring (Urvoy et al., *in prep*.). Surface waters were collected at nine stations covering the salinity gradient (Fig. 1). Station 1 (S1) was a freshwater reference site upstream of the dam and was not subjected to tidal influence. Station 2 (S2) corresponded to a freshwater station (salinity 0) under tidal influence and was collected at a fixed location in front of the dam. Stations 3–8 (S3–S8) were sampled every 5 units of salinity (5 to 30), monitored using a Cond330i thermosalinometer (WTW, Weilheim, Germany). Their location consequently varied depending on tides and river discharges. Finally, Station 9 (S9) was a marine reference sampled at the Service d'Observation en Milieu Littoral (SOMLIT, https://www.somlit.fr/) station of Saint Anne-du-Portzic (48°21′32.17″ N, 4°33′07.21″ W, salinity range: 33.3–34.7). Sampling was carried out from high to mid-tide. The marine station was first sampled and processed immediately. Sampling was then carried out from S1 to S8 within 4 h.

### Physicochemical variables

Temperature and salinity were measured in situ using a Cond330i thermosalinometer (WTW). Dissolved inorganic nitrogen (DIN), phosphate, silicate, dissolved organic phosphorus (DOP), suspended particulate matter (SPM), particulate inorganic matter (PIM), total particulate phosphorus (TPP), particulate inorganic phosphorus (PIP), chlorophyll *a* (Chl*a*), and pheopigments (Pheo) were measured as described in the Supplementary methods. Particulate organic matter (POM) was determined as the difference between SPM and PIM and expressed as a percentage of SPM (%POM). Particulate organic phosphorus (POP) was determined as the difference between TPP and PIP and expressed as a percentage of TPP (%POP). The percentage of Chl*a* (%Chl*a*) was determined as Chl*a* over Chl*a* and Pheo.

### DNA sequencing and bioinformatics

The BCC was determined using metabarcoding of the V3-V4 region of the 16S rRNA gene. PA communities were recovered using 3-µm filters (Whatman Nuclepore PC membrane). Several filters were used per station depending on the turbidity to avoid clogging. The 3-µm filtrates were filtrated through 0.2-µm filters (Whatman Nuclepore PC membrane) to recover the FL communities. Filters were flash-frozen in liquid nitrogen and stored at − 80 °C until processing. Blank dry filters were sampled simultaneously and used as contamination controls. DNA was extracted using the NucleoSpin Plant II Kit (Macherey–Nagel Ref. 740770.50) as advised by the supplier with an additional lysis step performed for 2 h at 56 °C with 25 mL of proteinase K (20 mg mL^−1^, Macherey Nagel Ref. 740506) and 100 mL of lysozyme (20 mg mL^−1^, Sigma ref 4403–5 g). If several filters were used for one station, the extracted DNA was pooled. The libraries were prepared (341F/785R primers^39^) and sequenced on the GénomeQuebec platform (Illumina MiSeq, 2 × 300 bp), resulting in 13,387,145 reads over 73 samples. The data were preprocessed using the SAMBA pipeline (v3.0.1, https://github.com/ifremer-bioinformatics/samba) implementing QIIME2^40^ (v2019.10.0). Briefly, reads were quality-trimmed and joined. Amplicon sequence variants (ASVs) were inferred and denoised using q2-dada2^41^. This resulted in 25,949 ASVs that were clustered using q2-dbOTU^42^, yielding 16,906 ASVs (35% clustering). The final ASVs were assigned against the Silva v138 database^43^ formatted for the 341F/805R primers, and a phylogenetic tree was built using fasttree in q2-phylogeny.

### Statistical analysis

All data were analyzed in R^44^ (v4.1.0) implemented in RStudio (v2021.09.0) and displayed using ggplot2^45^ (v3.3.5) unless specified otherwise. The correlations between environmental variables were visualized using a principal component analysis (PCA) on z-score standardized variables (FactoMineR^46^ package, v2.4). Spearman rank coefficients (stats package^44^, v4.1.10) were computed on raw data and displayed using the corrplot package^47^ (v0.90).

Metabarcoding data were analyzed using the phyloseq^48^ (v1.36.0), vegan^49^ (v2.5-7), and microeco^50^ (v0.6.0) packages. The ASVs corresponding to eukaryotes, archaea, mitochondria, and chloroplasts were removed (22% of total reads, 15,911 remaining ASVs). The rarefaction curves (ranacapa^51^, v0.1.0) revealed that all samples reached saturation (mean: 49,765 reads sample^−1^, range: 11,922–82,219 reads sample^-1^) (Figure S1). Linear discriminant analysis (LDA) effect size (LefSe) was performed using a LDA score cutoff of 1.5 and a p-value cutoff of 0.05 (microbiomeMarker^52^, v1.1.1).

Species richness, Shannon index, and Faith's phylogenetic diversity (PD) α-diversity indices were computed (phyloseq and PhyloMeasures^53^, v2.1) on rarefied counts (phyloseq, rarefaction depth = 26,700 reads per sample, rngseed = 999), and differences were tested using the Wilcoxon test (ggpubr). Rarefaction resulted in the removal of two samples (PA communities in S9 of April 2019 and S9 of July 2020) (Figure S1).

Two β-diversity indices were computed on the read count table transformed to relative abundances (total sum scaling)^54^. Bray–Curtis dissimilarity (phyloseq) considers the ASV abundance and ranges from 0 (no dissimilarity) to 1 (complete dissimilarity). The abundance-weighted β-mean nearest taxon distance (βMNTD) (microeco) considers the mean pairwise phylogenetic distance between each ASV in one community and its closest relative in the second community, weighted by the ASV abundance. β-diversity was visualized using principal coordinate analysis (PCoA, ape^55^, v5.5) applying the Cailliez correction for negative eigenvalues^56^. Permutational multivariate analysis of variance (PERMANOVA) was performed, and variance homogeneity was checked (*adonis2* and *betadisper* test with the Cailliez correction, respectively, vegan package).

The community assembly processes were assessed in the framework developed by Stegen et al*.*^57,58^ based on phylogenetic null modeling. Briefly, the abundance-weighted βMNTD was calculated for all pairwise comparisons of samples. The null βMNTD distribution was quantified by randomly shuffling the ASV name and abundance across the tips of the phylogenetic tree (999 permutations, microeco). The β-nearest taxon index (βNTI) represents the number of standard deviations that βMNTD departs from the mean of the null distribution. A βNTI > 2 or < − 2 indicates that the community assembly is governed by deterministic processes (variable and homogeneous selection, respectively). A βNTI between − 2 and 2 indicates the predominance of stochastic processes. For this range of βNTIs, the Bray–Curtis-based Raup-Crick score (RC_Bray_) was estimated (999 permutations, microeco). An RC_Bray_ < − 0.95 suggests the predominance of homogenizing dispersal, an RC_Bray_ > 0.95 suggests dispersal limitation, and an RC_Bray_ between − 0.95 and 0.95 can be interpreted as drift or undominated mechanism^57^.

Cohesion, a measure of the degree of connectivity of a community, was calculated for all samples as proposed by Herren and McMahon^28^. Briefly, the pairwise Pearson correlation of all ASVs was computed and corrected by the "expected" correlation generated from null modeling. The average positive and negative corrected correlations were calculated for each ASV (connectedness metric). The positive and negative cohesion values were computed for each sample by summing the abundance-weighted positive and negative connectedness metrics, thus ranging between − 1 to 0 and 0 to 1, respectively. The absolute value of cohesion increases when the abundances of highly connected taxa increase, reflecting the degree of correlation or connectivity within a community^28^. The cohesion metrics were computed using the authors' script (https://github.com/cherren8/Cohesion; persistence cutoff = 0.1; number of iterations = 200; shuffle algorithm = taxa shuffle). As per author guidelines, low-prevalence ASVs (comprising fewer than 150 reads across all samples) were removed beforehand, removing most ASVs (1959 or 12.3% remaining ASVs) but retaining most reads (92.1% remaining reads).

The effect of cohesion and abiotic variables on the composition of the FL and PA communities was assessed using distance-based redundancy analysis (dbRDA) and variance partitioning on the Bray–Curtis dissimilarity (with the Cailliez correction, vegan). The absolute value of negative cohesion was used so that the amount of negative correlations increased with the absolute value of negative cohesion. All predictors were z-score standardized, and multicollinearity was checked using the variance inflation factor (VIF, usdm^59^, v1.1-18). DIN, silicate, and %Chl*a* were thus removed (final VIF < 10). The explained variance was adjusted and the model and axis significance was tested (anova.cca, vegan, 1000 permutations). The effect of cohesion and abiotic variables on the FL-PA pairwise dissimilarity was estimated using redundancy analysis (RDA, vegan) on Bray–Curtis dissimilarity and βMNTD, as described for dbRDA. DIN, silicate, %Chl*a*, and PA negative cohesion were removed (final VIF < 10).

## Results

### Physicochemical characteristics

**Figure 2:**
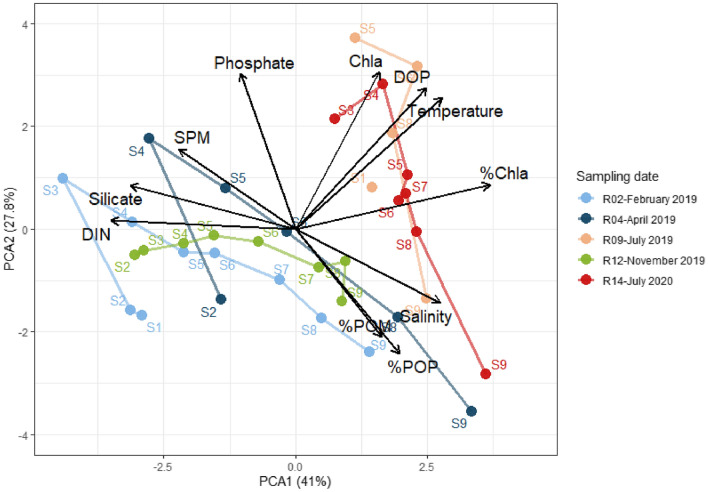
Principal component analysis (PCA) representing the physicochemical characteristics of all samples. The sampling station is indicated for each sample. The lines link the different stations within one sampling time in their spatial order (S1–S9).

Physicochemical variable values are shown in Table S1 and plotted in Figure S2. The temperature varied from 6.3 to 23.4 °C throughout the samplings. In February and November, the river end (S1) was colder than the marine end (S9), while the opposite trend was observed on the other sampling dates. DIN and silicate followed the theoretical dilution curve along the salinity gradient (respective Spearman correlation of − 0.93 and − 0.97 with salinity, Figures S2, S3), and both decreased during summer throughout the estuary. In contrast, phosphate did not follow the theoretical dilution curve but increased in S3–S8 compared to S1 and S9, and was higher in July 2019 than on the other sampling dates (Figure S2). DOP did not follow a clear pattern along the estuary and increased in summer (July 2019, 2020) compared to the other seasons. SPM increased between salinity 5 and 10 (S3–S4), which corresponded to the maximum turbidity zone (MTZ). SPM was especially important in February and April 2019, reaching 197 and 112 mg L^−1^, respectively. The MTZ (S3–S4) and downstream estuarine stations (S5–S8) were characterized by a lower %POM and %POP, consistent with the presence of less labile particles compared to the freshwater and marine stations. Chl*a* and %Chl*a* increased in summer (Figs. 2, S2), concomitantly with the increase in temperature and decrease in turbidity that favored light penetration and phytoplankton growth. Consequently, the samplings were differentiated based on salinity and sampling season, with a clear difference between the summer samplings (July 2019, 2020) and the other samplings (Fig. 2).

### Spatiotemporal variations in the FL and PA bacterial communities

#### Bacterial community composition

**Figure 3:**
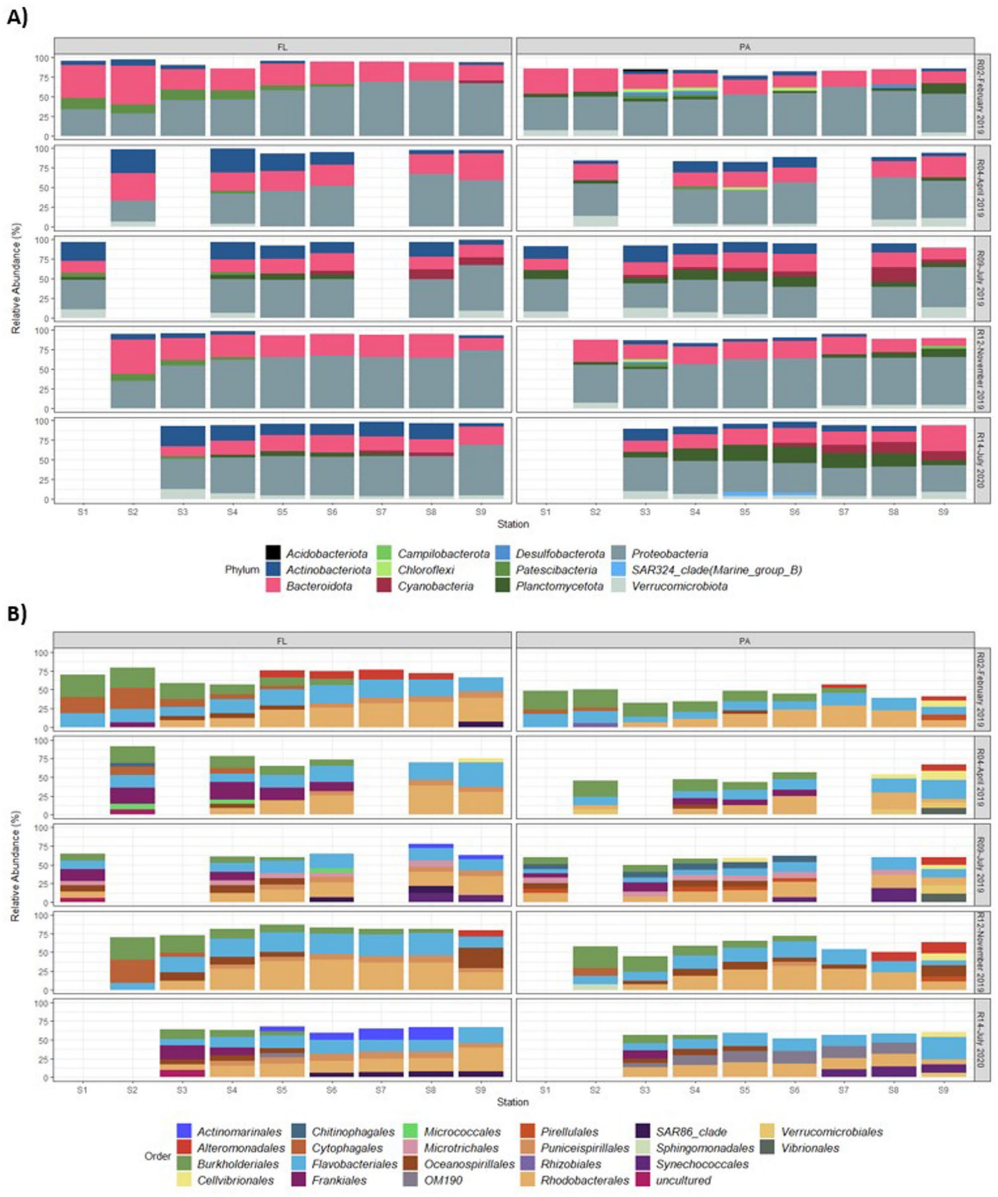
Bar plots showing the taxonomic composition of the FL and PA bacterial communities, aggregated at the phylum (**A**) or order (**B**) rank. Only the groups representing more than 3% (phylum) or 5% (order) within a sample are represented for readability.

Overall, the bacterial communities were dominated by *Proteobacteria* (mean relative abundance and standard deviation of 50 ± 11% across all samples), *Bacteroidia* (22 ± 7% rel. abundance), *Actinobacteriota* (9 ± 8% rel. abundance), *Planctomycetota* (5 ± 5% rel. abundance) and *Verrucomicrobia* (4 ± 3% rel. abundance). The BCC presented a continuous shift along the salinity gradient (Fig. 3). Lefse showed that the most abundant groups enriched in the freshwater stations (S1–S2) were *Burkholderiales* (23 ± 8% rel. abundance, *Betaproteobacteria*) and *Cytophagales* (11 ± 11% rel. abundance, *Bacteroidota*) (Figure S4). In contrast, the most abundant group enriched in the MTZ (S3–S4) was *Frankiales* (7 ± 7% rel. abundance, *Actinobacteriota*). At the downstream estuarine stations (S5–S8), *Rhodobacterales* (24 ± 7% rel. abundance, *Alphaproteobacteria*), *Flavobacteriales* (18 ± 5% rel. abundance, *Bacteroidota*) and *Puniceispirillales* (5 ± 2% rel. abundance, *Alphaproteobacteria*) were enriched. Finally, the marine stations (S9) were enriched in various *Gammaproteobacteria*, such as *Oceanospirillales* (6 ± 8% rel. abundance), *Cellvibrionales* (6 ± 3% rel. abundance) and *Alteromonadales* (5 ± 5% rel. abundance) (Fig. 3B).

Differences were also observed between the PA and FL fractions in summer (July 2019, 2020) and in the other seasons (February, April and November 2019) throughout the estuary (Figure S5). In summer, the FL communities were enriched in *Puniceispirillales* (7 ± 2% rel. abundance, *Alphaproteobacteria*) and *Actinomarinales* (6 ± 6% rel. abundance, *Actinobacteriota*), while the PA were enriched in OM190 (7 ± 7% rel. abundance, *Planctomycetota*) and *Synechococcales* (6 ± 6% rel. abundance, *Cyanobacteria*) but also in less abundant orders usually involved in carbohydrate degradation (e.g., *Chitinophagales*, *Verrucomicrobiales*, *Planctomycetales*). In the other seasons, the FL communities were mainly enriched in *Burkholderiales* (12 ± 9% rel. abundance, *Betaproteobacteria*), *Flavobacteriales* (21 ± 6% rel. abundance, *Bacteroidota*) and *Cytophagales* (6 ± 9% rel. abundance, *Bacteroidota*). In contrast, the PA communities were mainly enriched in *Alteromonadales* (3 ± 4% rel. abundance, *Gammaproteobacteria*) and *Rhizobiales* (2 ± 1% rel. abundance, *Alphaproteobacteria*), and to a lesser extent in orders containing anaerobic bacteria present in marine sediments (*Anaerolineales*, *Desulfobulbales*, *Desulfobacteriales*).

#### α-Diversity

**Figure 4:**
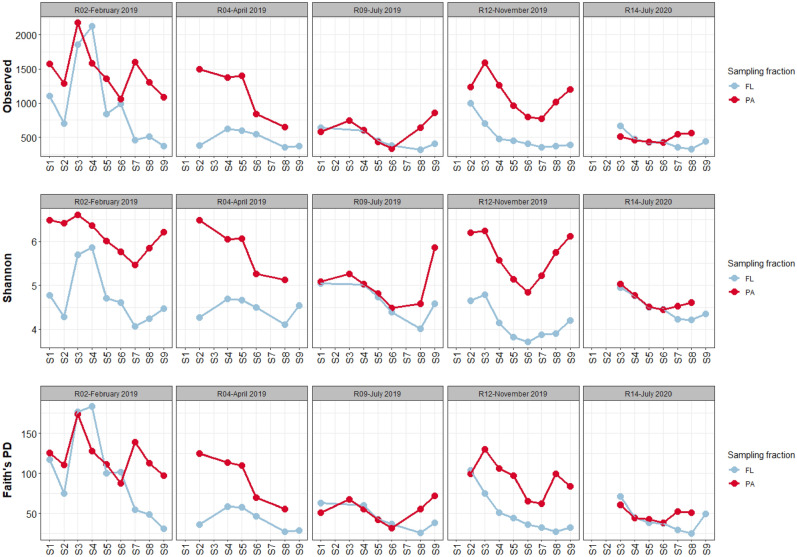
Alpha diversity indices (richness, Shannon index and Faith's PD) characterizing the PA and FL communities along the Aulne estuary salinity gradient at the five sampling dates. Two samples were removed in the rarefication process (PA community in S9 of April 2019 and S9 of July 2020).

All three α-diversity indices exhibited similar patterns (Fig. 4). In February, April and November 2019, there was a higher α-diversity in the PA fraction (mean richness: 1256; Shannon: 5.9; Faith's PD: 104) than in the FL fraction (mean richness: 694; Shannon: 4.5; Faith's PD: 67) (Wilcoxon, *p* < 0.001 for all indices). In contrast, both fractions exhibited similar α-diversity levels in summer (July 2019, 2020) (Wilcoxon, not significant except for the Shannon index: mean PA = 4.8, mean FL = 4.6, *p* < 0.05). The PA community α-diversity decreased in summer (July 2019, 2020) (Fig. 4), and thus, all α-diversity indices were negatively correlated with temperature (richness: R^2^_adj_ = 66%, Shannon: R^2^_adj_ = 58%, Faith’s PD: R^2^_adj_ = 65%; *p* < 0.001 for all indices). In contrast, the α-diversity of the FL communities was relatively stable over time (Fig. 4); temperature thus explained less variance (richness: R^2^_adj_ = 17%, *p* < 0.001; Shannon: not significant; Faith’s PD: R^2^_adj_ = 20%, *p* < 0.001). Along the salinity gradient, the α-diversity of the FL and PA communities was higher upstream (S1–S4) and in marine waters (S9) than in the intermediate stations (S5–S8) (Wilcoxon, *p* < 0.05 for all except PA communities’ richness), although these variations were less pronounced for the FL communities.

#### β-Diversity

**Figure 5:**
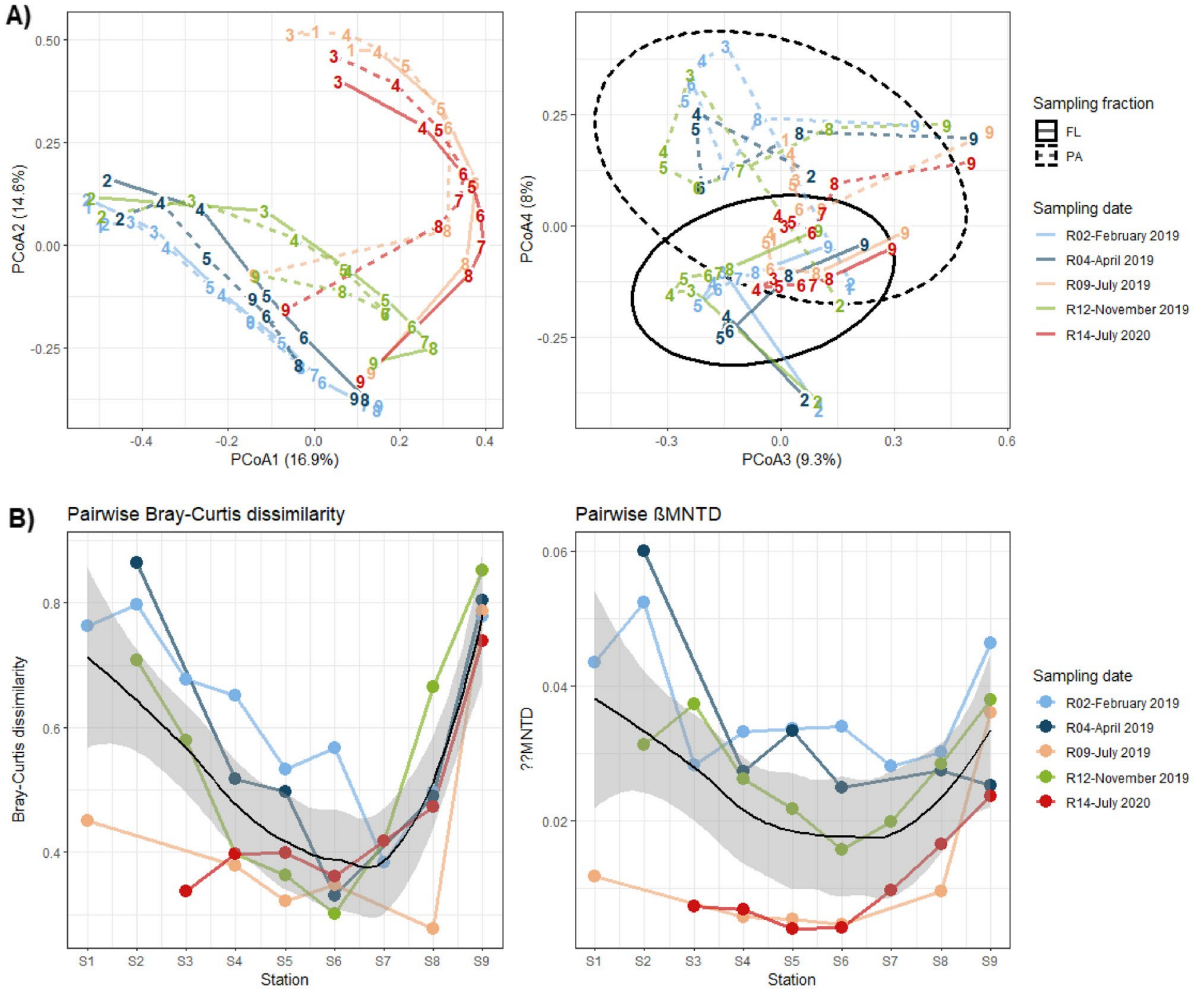
**(A)** PCoA ordination of all samples based on the Bray–Curtis dissimilarity (left: axes 1–2; right: axes 3–4). The lines link the different stations within one sampling time in their spatial order (S1–S9) (solid line: FL communities, dashed line: PA communities). Samples are labeled as stations. **(B)** Pairwise Bray–Curtis and abundance-weighted βMNTD dissimilarity between the FL and PA fractions for each sample. The black line represents the loess regression curve and the dark area represents its confidence interval.

The Bray–Curtis-based (taxonomic composition) PCoA showed a marked spatial pattern with a separation between the freshwater (S1–S2) and marine samples (S9) along the 1st axis (16.9% of variance) (Fig. 5A, left). There was also a clear seasonal pattern with the 2nd axis (14.6% of variance) separating the summer samples (July 2019, 2020) from the other seasons. The 3^rd^ axis (9.3% of variance) opposed the end-stations (S1–S2, S9) to the intermediate estuarine stations (S3–S8) (Fig. 5A, right). The communities also differed according to the sampling fraction (4th axis, 8.0% of variance). Coherently, most of the variance was explained by the sampling station (PERMANOVA, 27%, F = 4.7, *p* < 0.001), sampling time (PERMANOVA, 25%, F = 8.8, *p* < 0.001) and sampling fraction (PERMANOVA, 7%, F = 9.5, *p* < 0.001). The Betadisper test showed no difference in dispersion for the sampling time and sampling fraction (*p* > 0.05), supporting that the PERMANOVA results came from a difference in centroid location. Significant heterogeneity in dispersion was found for the sampling stations (*p* < 0.05); however, the PCoA plot clearly supported a difference in both centroid location and dispersion.

The first two axes of the PCoA based on abundance-weighted βMNTD (phylogenetic composition) showed a similar pattern overall but explained less variance, highlighting complex variations in the phylogenetic β-diversity (Figure S6). Part of the variance was explained by the sampling station (PERMANOVA, 16%, F = 1.8, *p* < 0.001), the sampling date (PERMANOVA, 11%, F = 2.3, *p* < 0.001) and the sampling fraction (PERMANOVA, 4%, F = 3.5, *p* < 0.001). The Betadisper test showed no difference in dispersion.

#### FL-PA pairwise dissimilarity

The PA and FL communities were always dissimilar, as highlighted by the pairwise Bray–Curtis dissimilarity values (mean: 0.53, range: 0.28–0.86). However, the FL-PA dissimilarity decreased in the mid-salinity range (S4–S8) compared to both the marine and freshwater stations (Wilcoxon, *p* < 0.01 for Bray–Curtis and βMNTD) (Fig. 5B). Interestingly, the FL-PA pairwise βMNTD, considering phylogeny, also decreased in summer (July 2019, 2020) compared to the other seasons (February, April, November 2019) (mean summer = 0.011; mean other seasons = 0.032; Wilcoxon, *p* < 0.001), which was especially pronounced at the S1–S8 stations. This pattern was present but less clear with taxonomic dissimilarity (mean summer = 0.44; mean other seasons = 0.58; Wilcoxon, *p* < 0.05).

### Bacterial community cohesion

**Figure 6:**
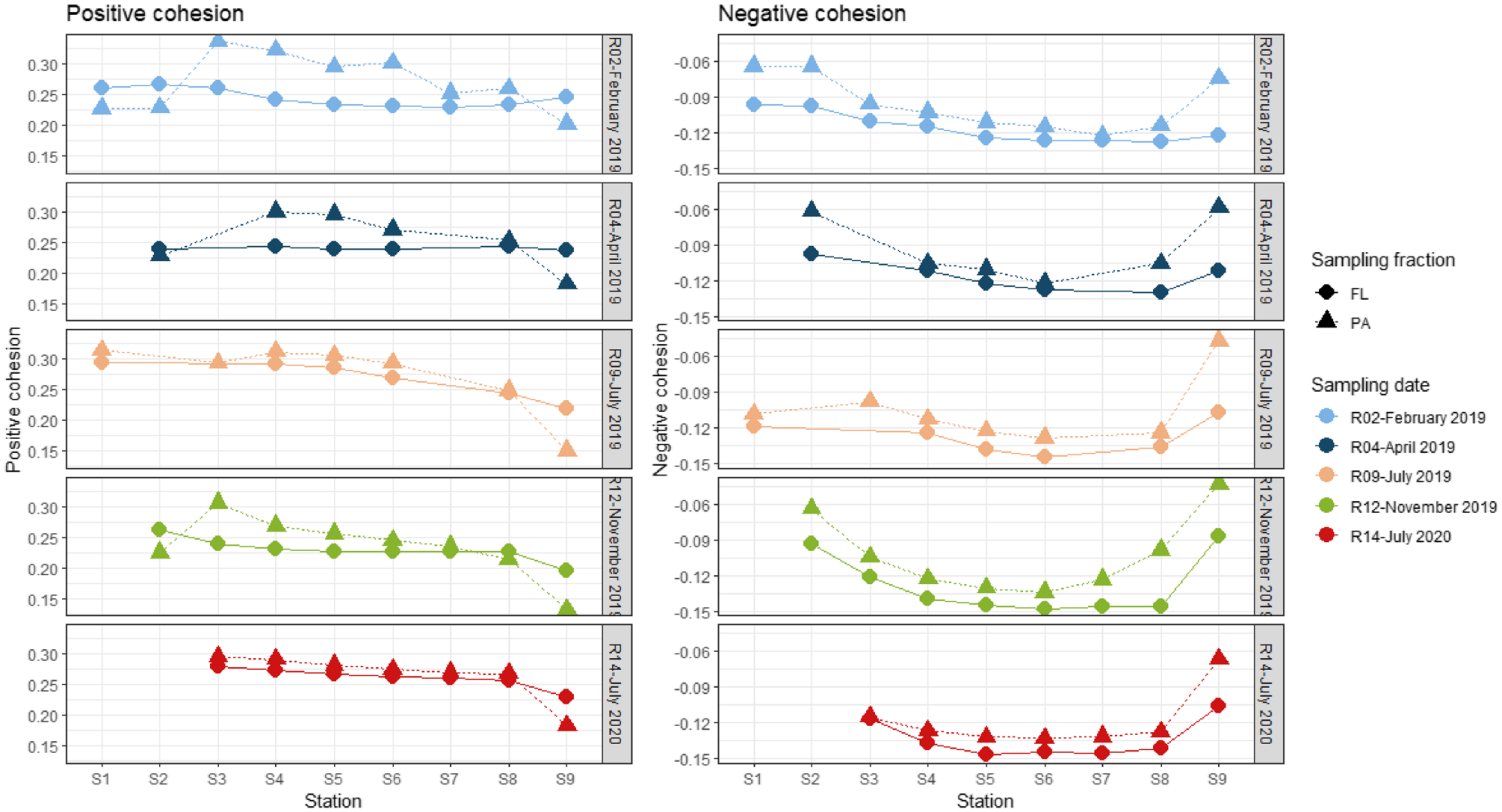
Positive and negative cohesion of FL and PA communities along the salinity gradient of the Aulne estuary.

The positive cohesion, reflecting the degree of positive correlations, was overall higher in the PA communities than in the FL communities (Wilcoxon, *p* < 0.05), especially at the intermediate stations (S3–S8) in February, April and November (Fig. 6). The FL positive cohesion decreased from S1 to S9 (range: 0.19–0.29). The PA positive cohesion was more variable (range: 0.13–0.34). It first increased in the MTZ (S3) (especially in February, April and November 2019), then decreased from S3 to S9, with a sharper decrease at the marine station (S9). Both FL and PA positive cohesion increased in the summer samplings, although it was not significant for the PA communities (Wilcoxon, FL: *p* < 0.001; PA: *p* = 0.22). The absolute value of negative cohesion, reflecting the degree of negative correlations, was more important in the FL fraction than in the PA fraction (Wilcoxon, *p* < 0.001). For both the FL and PA communities, the absolute negative cohesion increased at the intermediate stations (S4–S8) compared to the end-stations (Wilcoxon, *p* < 0.001). Both FL and PA absolute negative cohesion tended to increase in the summer samplings, although it was not significant for the FL communities (Wilcoxon, FL: *p* = 0.16; PA: *p* < 0.05).

### Community assembly processes

All FL and PA community assemblies were largely dominated by deterministic homogeneous selection (βNTI < − 2, Figure S7), with a mean contribution of 96% to assembly processes. In February, April and November 2019, PA communities were more significantly impacted by homogeneous selection than FL communities, as the mean βNTIs were significantly lower (Wilcoxon, *p* < 0.005). In contrast, in the summer group, the mean PA community's βNTI was either not significantly different (July 2019) or significantly higher (July 2020) than the mean FL-βNTIs. Stochastic processes also contributed to the FL community assembly by 16.7% (2.8% dispersal limitation and 13.9% undominated) and 14.3% (8.3% homogenizing dispersal and 2.8% undominated) in February 2019 and November 2019, respectively.

### Biotic and abiotic factors driving the FL and PA community compositions

**Figure 7:**
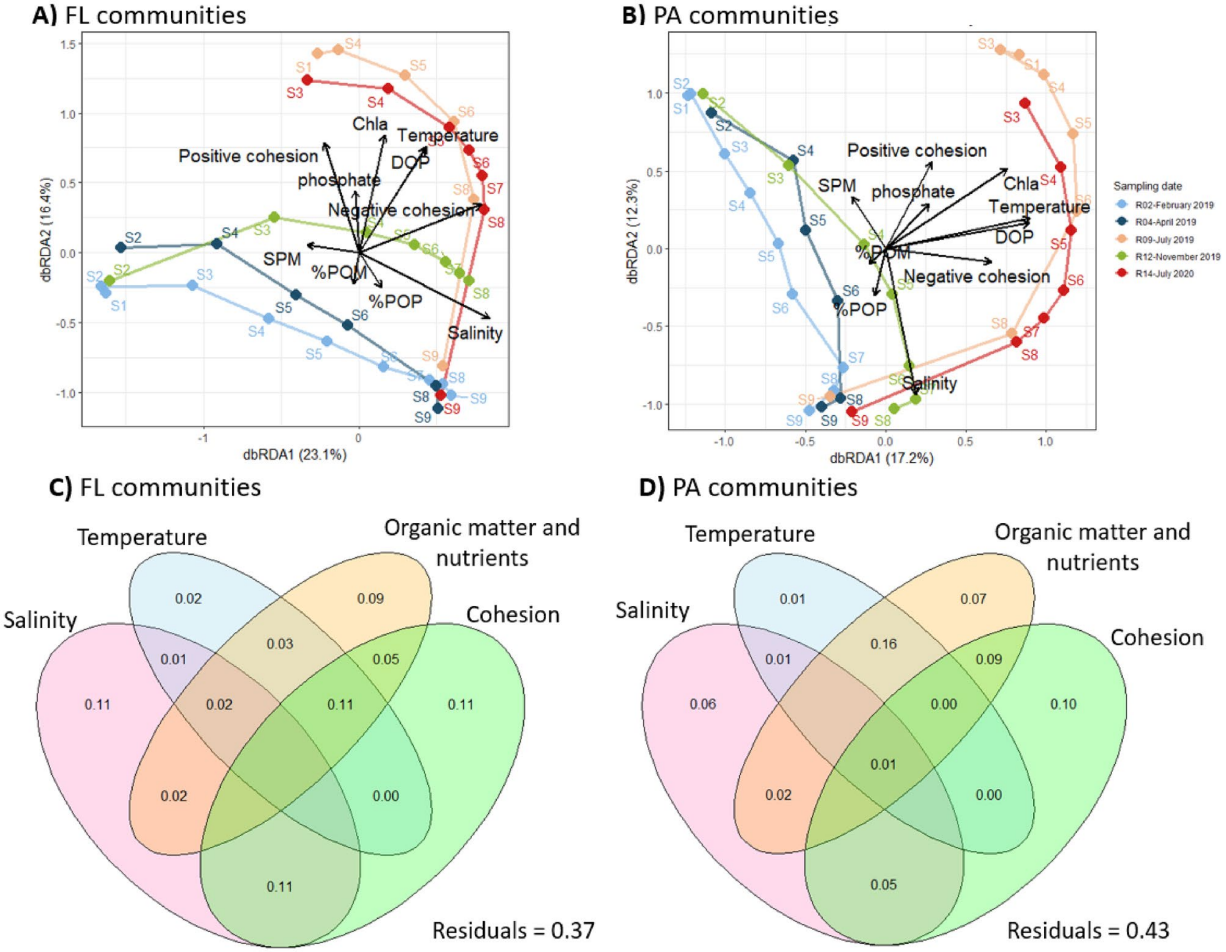
Bray–Curtis-based dbRDA (**A, B**) and variance partitioning (**C, D**) of the Bray–Curtis dissimilarity for the FL (**A–C**) and PA (**B–D**) communities. (**A, B**) The lines link the different stations within one sampling time in their spatial order (S1–S9) (solid line: FL communities, dashed line: PA communities). It should be noted that the absolute value of negative cohesion was used so that the degree of negative correlation increases with the absolute value of negative cohesion. The sampling station is indicated for each sample. (**C, D**) Organic matter and nutrients regroup the variables SPM, %POM, Chl*a*, DOP and phosphate, while cohesion comprises both positive and negative cohesion. Negative R^2^ values are not shown.

The dbRDA performed on the Bray–Curtis dissimilarity explained most of the variance for the FL (R^2^_ajd_ = 63%, *p* < 0.001) and PA communities (R^2^_ajd_ = 57%, *p* < 0.001) (Fig. 7). For both communities, the first two axes clearly separated the sampling stations along the salinity gradient (FL community: 1st axis, 23.1% of variance; PA community: 2nd axis, 12.3% of variance) (Fig. 7A, B). They also separated the summer samplings (July 2019, 2020) from the other sampling dates (February, April, November 2019) (FL community: 2nd axis, 16.3% of variance; PA community: 1st axis, 17.2% of variance). The summer samplings were correlated with seasonal factors such as temperature, DOP or Chl*a* but also with positive and negative cohesion. Variance partitioning showed that salinity, nutrients and organic matter, and cohesion explained similar amounts of variance on their own (FL: 11%, 9% and 11%, respectively; PA: 6%, 7% and 10%, respectively) (Fig. 7C, D). Temperature alone explained low amounts of variance (FL: 2%; PA: 1%), but an important part was shared with cohesion and/or organic matter and nutrients, reflecting their complex relationships.

### Biotic and abiotic factors driving the FL-PA dissimilarity

**Figure 8:**
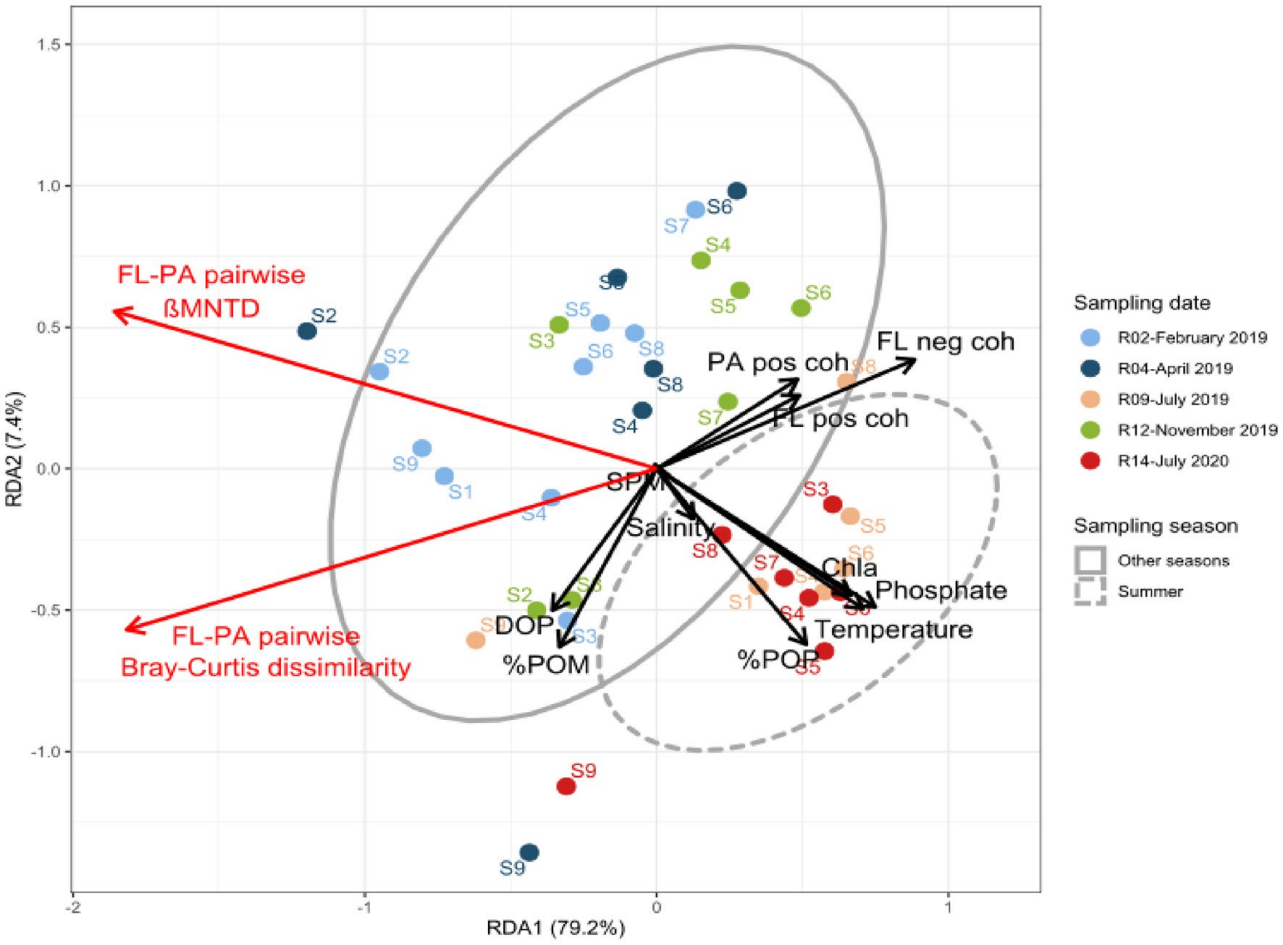
Redundancy analysis (RDA) of the pairwise FL-PA dissimilarity based on Bray–Curtis dissimilarity and βMNTD. The ellipses represent the sampling season (dashed line: summer, solid line: other seasons). It should be noted that the absolute value of negative cohesion was used so that the degree of negative correlation increases with the absolute value of negative cohesion. The sampling station is indicated for each sample. Pos: positive; neg: negative; coh: cohesion.

The RDA (R^2^_ajd_ = 76%, *p* < 0.001) performed on the pairwise FL-PA Bray–Curtis dissimilarity and βMNTD showed that the increased dissimilarity in the end-stations (S1, S9) was correlated with markers of particle lability (%POM, %POP), while the decreased dissimilarity in the estuarine stations was correlated with phosphate, PA positive cohesion and FL negative cohesion (Fig. 8). The decreased phylogenetic pairwise βMNTD in summer was correlated with seasonal variables (e.g., temperature, DOP, Chl*a*) but also with FL positive cohesion.

## Discussion

Characterizing FL and PA bacterial communities and their determinants in estuaries is a first step toward understanding their biogeochemical impact on the organic matter and nutrients exported to coastal ecosystems. Understanding the differences between FL and PA communities is especially interesting since they contribute differently to ecosystem functioning. This study depicted, for the first time, the diversity, composition and driving factors of the FL and PA bacterial communities within the macrotidal Aulne estuary and the adjacent Bay of Brest coastal waters.

### FL and PA assembly processes were dominated by homogeneous selection

Ecological null modeling showed that FL and PA bacterial community assemblies were largely driven by homogeneous selection, indicating that the biotic and abiotic environmental conditions led to bacterial communities that were more similar than what would be expected by random changes. Few studies have quantified assembly processes for FL and PA communities in coastal ecosystems; however, homogeneous selection was an important driver in other land–sea transition areas^60–62^. The predominance of deterministic processes has been suggested to lead to communities being well adapted to their environment, thus potentially having a stronger biogeochemical impact than communities assembled through stochastic processes^63^. This result is consistent with the idea of estuaries being natural bioreactors where organic matter is heavily transformed by estuarine bacteria^32^.

### FL and PA communities followed pronounced spatiotemporal patterns driven by similar factors

The β-diversity of the FL and PA communities differed largely across space and time and was driven by similar variables with comparable relative importance. Since the sampling stations and sampling time explained most of the variance in the FL and PA communities' β-diversity (Fig. 5 and associated PERMANOVA), the salinity and seasonal variables (e.g., temperature, Chl*a*, DOP) explained a large part of the bacterial community variation (Fig. 7). Salinity is logically an important driver of bacterial communities along fresh to marine water gradients such as fjords, estuaries and coastal margins^16,19,22,23,64,65^*.* Salinity reflects the mixing of freshwater and marine water masses and directly impacts the BCC by selecting taxa capable of growing at a specific saline concentration. Similar to other studies^16,22,64^, the freshwater communities shifted from a predominance of *Actinobacteriota* (e.g., *Frankiales*) and *Betaproteobacteria* (e.g., *Burkholderiales*) to a majority of *Alphaproteobacteria* (e.g., *Rhodobacterales*, *SAR11*), *Gammaproteobacteria* (e.g., *Alteromonadales*, *Oceanospirillales*, *Vibrionales*) and *Bacteroidetes* (e.g., *Flavobacteriales*) in the estuarine and marine samples. However, the BCC in the estuarine stations (S3–S8) did not result only from the mixing of water masses: these stations hosted native FL and PA bacterial communities that differed from both the freshwater (S1–S2) and the marine (S9) stations (Fig. 5A, right panels). A native estuarine community can occur only if the doubling time of the bacteria within the estuary is smaller than the flushing time of the water. This is the case in the Aulne estuary, where the bacterial generation times estimated from bacterial production were extremely fast for all sampling times (11–55 h and 1–12 h for the FL and PA communities, respectively, for S3–S8; Urvoy et al*.*, *in prep*.) compared to the residence time of the water masses (3–30 days^38^). This reinforces the idea that the Aulne estuary is a biogeochemical bioreactor hosting adapted and active specific communities.

Among seasonal variables, water temperature is an important driver of microbial communities and can directly influence BCC and metabolic activity^66,67^. However, temperature on its own explained low amounts of variance, as it was correlated with other variables, such as Chl*a*, %Chl*a*, DOP, and phosphate (Fig. 7), which can select for specific bacterial taxa. For instance, *Flavobacteria* (*Bacteroidota*), members of the Roseobacter clade (*Rhodobacterales*, *Alphaproteobacteria*) and *Gammaproteobacteria* (e.g., *Alteromonadaceae*) have been consistently linked with algal bloom occurrences^68^. In our study, the communities in summer largely differed from the communities in the other seasons. They were less diverse, enriched in autotrophic *Synechoccocales* and groups usually involved in carbohydrate degradation (*Chitinophagales*, *Verrucomicrobiales* or *Planctomycetales*)^69,70^. The latter can be linked the high Chl*a* and %Chl*a* values measured in summer within the estuary, suggesting the presence of phytoplankton likely impacting both DOM and POM pools.

In addition to variables related to space and time, cohesion explained an important fraction of the variance on its own as well as shared with organic matter, temperature or salinity (Fig. 7). This suggested the importance of cohesion in explaining BCC patterns and its link with environmental variables. Negative cohesion reflects the importance of negative co-occurrences, which can result from differences in niches or antagonist interactions such as competition (i.e., the taxa compete for the same resource), among others^71,72^. In contrast, positive cohesion reflects the magnitude of positive co-occurrences, which can result from beneficial interactions (e.g., division of labor to exploit the same resource) or niche partitioning^71,72^. As such, even though they are not appropriate for implying accurate interactions^72^, co-occurrence patterns can drive community evolution by creating feedback loops that can stabilize or differentiate the communities^71,72^. Consequently, cohesion was found to be an important factor explaining BCC in other studies^28,73,74^. In our study, the summer samples contained higher positive and negative cohesion levels than did the other sampling dates, possibly reflecting their importance in structuring the community from winter to summer.

### FL-PA dissimilarity differed across space and time and was driven by various factors

Even though the sampling fraction explained less variance than the sampling station and date, the FL and PA communities were largely dissimilar (Fig. 5). The pairwise FL-PA Bray–Curtis dissimilarity (taxonomic composition) and βMNTD (phylogenetic composition) varied spatially and temporally. Most strikingly, both FL-PA taxonomic and phylogenetic dissimilarities decreased in the intermediate estuarine stations compared to the end-stations for all sampling dates. The pairwise βMNTD also showed that FL-PA dissimilarity significantly decreased in summer (July 2019, 2020) compared to the other seasons throughout the estuary, which was concomitant with a decrease in PA α-diversity. These patterns can be explained by the superposition of several factors (Fig. 8). The higher FL-PA dissimilarity in the freshwater and marine stations (S1–S2, S9) was correlated with the presence of more labile POM compared to the inner estuarine stations, as suggested by higher %POM and %POP. Other studies have also shown that the marine station (S9) POM pool was be largely dominated by phytoplankton^75^, while the presence of numerous weirs favored phytoplankton development in the freshwater station (S1)^76^. The colonization of labile POM was suggested to provide a larger advantage than the colonization of refractory terrestrial POM commonly found in estuaries, thus increasing the FL-PA dissimilarity^15,16^. Conversely, the estuarine stations contained less labile POM, possibly explaining the lower FL-PA dissimilarity. Estuary are known to contain refractory POM that originates from terrestrial run-off, which is further transformed by physicochemical (e.g., flocculation, photoalteration) and microbial processes, favored by numerous sedimentation/resuspension cycles^33,34^. In addition, these estuarine stations were characterized by higher PA positive cohesion and FL negative cohesion levels. The increased PA positive cohesion could suggest that colonizing bacteria cooperated to degrade complex, refractory estuarine POM. Interestingly, SPM did not seem to explain the FL-PA dissimilarity (Fig. 8). However, they may contribute to the FL-PA dissimilarity in February, April and November 2019, when strong hydrodynamic forcings may resuspend benthic communities. This would be consistent with the enrichment of anaerobic bacterial groups usually found in marine sediments (*Anaerolineales*, *Desulfobulbales*, *Desulfobacteriales*) on these sampling dates.

The decrease in phylogenetic FL-PA dissimilarity in summer was logically correlated with seasonal variables (temperature, Chl*a*, DOP) and FL positive cohesion. POM and DOM are expected to be more labile in summer since autochthonous processes (e.g., primary production) increase compared to allochthonous inputs (e.g., refractory terrestrial matter run-off), which is suggested by increased Chl*a* and %Chl*a* in summer (July 2019, 2020). Consistently, a different study led in the Aulne estuary has reported a decreased particulate organic carbon to nitrogen ratio in summer (6.6 ± 0.3) compared to winter, where high values (8.6 ± 0.1) were reflective of terrestrial inputs^77^. In particular, algal components were shown to account for 65–80% of POM in summer, compared with 5% in the other seasons^77^. As such, these results seem conflicting: on the one hand, more labile POM was linked to a higher taxonomic and phylogenetic dissimilarity in the end-stations; on the other hand, FL-PA phylogenetic dissimilarity decreased in summer, which most likely contains more labile organic matter. A possible explanation is that the autochthonous labile DOM and POM produced during summer have similar molecular compositions (e.g., phytoplankton components such as polysaccharides or proteins). This may lead to the selection of closely related taxa able to assimilate similar substrates in both fractions and thereby increase the communities' positive cohesion (e.g., increase in positive co-occurrence driven by similar niches). Simultaneously, PA communities are known to solubilize POM into DOM through their extracellular hydrolysis enzymes^6,78^. The resulting DOM can be assimilated by FL bacteria, likely shaping their BCC and favoring co-occurrence patterns between the two compartments. It is possible that these DOM releases increase in summer due to a higher bacterial metabolism, especially within the estuary (Urvoy et al*.*, *in prep*.). This would likely participate in the homogenization of substrate pools and reduce the FL-PA dissimilarity. The decreased river flow in summer also likely favors the exchange of bacteria and organic matter between both fractions by increasing particles and water residence time.

## Conclusion

This study depicted the FL and PA bacterial communities within a temperate macrotidal estuary, the Aulne estuary, for the first time, a step toward understanding their biogeochemical impact. It showed the importance of deterministic processes in structuring both the FL and PA communities along the salinity gradient. Their compositions were driven by salinity and seasonal variables as well as by cohesion, highlighting the need to integrate co-occurrence patterns in such studies. Interestingly, our study showed that FL-PA dissimilarity varied across space and time, and that taxonomic and phylogenetic dissimilarity painted complementary pictures. Given the large taxonomic differences between the FL and PA communities, it is likely that they differ in their metabolic capacities and possibly differentially affect the inputs to the Bay of Brest, although future studies are needed to investigate this hypothesis further.

## Supplementary Information

Supplementary Information.

## Data Availability

The sequencing data generated during this study are available on online repositories under the accession number PRJNA825348.
