# Supplementary material for: Free-living and particle-attached bacterial community composition, assembly processes and determinants across spatiotemporal scales in a macrotidal temperate estuary
Source: Sci Rep. 2022 Aug 16;12:13897. doi: 10.1038/s41598-022-18274-w (PMC9381549; doi:10.1038/s41598-022-18274-w)
Supplement: Supplementary file 1 — Supplementary Information. [file 41598_2022_18274_MOESM1_ESM.pdf]

Free-living and particle-attached bacterial community composition, assembly processes and determinants across spatiotemporal scales in a macrotidal temperate estuary

Marion Urvoy[1,2,*], Michèle Gourmelon[1], Joëlle Serghine[1], Emilie Rabiller[1], Stéphane L'Helguen[2], Claire Labry[1,*]

[1]Ifremer, DYNECO, F-29280 Plouzané, France

[2]Université de Bretagne Occidentale, CNRS, IRD, Ifremer, UMR 6539, Laboratoire des Sciences de l'Environnement Marin (LEMAR), F-29280 Plouzané, France

[*]Correspondence: marion.urvoy@outlook.fr, claire.labry@ifremer.fr

Running title:

Bacterial community composition in a macrotidal estuary

Keywords:

Bacteria, free-living, particle-attached, bacterial community composition, assembly processes, Aulne estuary, Bay of Brest (France)

I. Supplementary methods

Temperature (T) and salinity (S) were measured *in situ* using a WTW thermosalinometer. Samples for ammonium, nitrite, nitrate, phosphate and dissolved organic phosphorus (DOP) were filtered by gravity using pre-combusted (4 h, 480°C) Whatman 25 mm GF/F glass-fiber filters. Filtrates were frozen at -20 °C until measurement. Samples for silicate were filtered (0.45 µm, cellulose acetate, Sartorius Minisart) and kept at 4°C. Samples for total particulate phosphorus (TPP) and particulate inorganic phosphorus (PIP), suspended particulate matters (SPM), chlorophyll *a* (Chl*a*) and pheopigments (Pheo) were filtered under low pressure (< 50 mm Hg) on pre-combusted Whatman GF/F filters and filters were stored at -20°C. Filtered volumes varied from 20 to 500 mL depending on turbidity.

The nutrient concentrations (ammonium, nitrite, nitrate and silicate) were measured using segmented flow analysis according to Aminot et al. (2009). TPP and PIP were determined as described in Labry *et al.* (2013) following the methods of Solorzano and Sharp (1980) and Aspila *et al.* (1976), respectively. DOP was analysed according to the alkaline persulfate oxidation procedure (Koroleff, 1983). These methods consist in converting the different phosphorus forms into phosphate, which was quantified with the phosphomolybdate-blue colorimetric reaction (Murphy and Riley, 1962) using either 5 cm cuve and Shimadzu UV 160 spectrophotometer (DOP and phosphate) or segmented flow analysis (TPP and PIP). Particulate organic phosphorus (POP) was determined as the difference between TPP and PIP and expressed in percentage of TPP (%POP). Dissolved inorganic nitrogen (DIN) was calculated as the sum of ammonium, nitrite and nitrate. SPM were measured using pre-weighted combusted GF/F filters. The filters were rinsed with Milli-Q water to remove salts. SPM corresponded to the increase in weight after drying at 70°C for 3 h. Particulate inorganic matter (PIM) corresponded to the weight after 2 h at 480°C. The particulate organic matter (POM) was determined as the difference between the two measurements and expressed in percentage of SPM (%POM). Chl*a* and Pheo were determined by acidification fluorometric procedure in 90% acetone extracts (Holm-Hansen *et al.*, 1965) and the percent of Chl*a* (%Chl*a*) was determined as Chl*a* over Chl*a* and Pheo.

References:

Aminot, A., Kérouel, R., and Coverly, S. (2009) Nutrients in seawater using segmented flow analysis. In Practical Guidelines for the Analysis of Seawater. Wurl, O. (ed). Boca Raton: CRC Press, pp. 143–178.

Aspila, K.I., Agemian, H., and Chau, A.S.Y. (1976) A semi-automated method for the determination of inorganic, organic and total phosphate in sediments. Analyst 101: 187–197.

Holm-Hansen, O., Lorenzen, C.J., Holmes, R.W., and Strickland, J.D.H. (1965) Fluorometric determination of chlorophyll. ICES J Mar Sci 30: 3–15.

Koroleff, F. (1983) Determination of phosphorus. In Methods of Seawater analysis. Grasshoff, K., Ehrhardt, M., and Kremling, K. (eds). Weinheim: Verlag Chemie, pp. 125–139.

Labry, C., Youenou, A., Delmas, D., and Michelon, P. (2013) Addressing the measurement of particulate organic and inorganic phosphorus in estuarine and coastal waters. Cont Shelf Res 60: 28–37.

Murphy, J. and Riley, J.P. (1962) A modified single solution method for the determination of phosphate in natural waters. Anal Chim Acta 27: 31–36.

Solorzano, L. and Sharp, J.H. (1980) Determination of total dissolved phosphorus and particulate phosphorus in natural waters. Limnol Oceanogr 25: 758–760.

II. Supplementary data

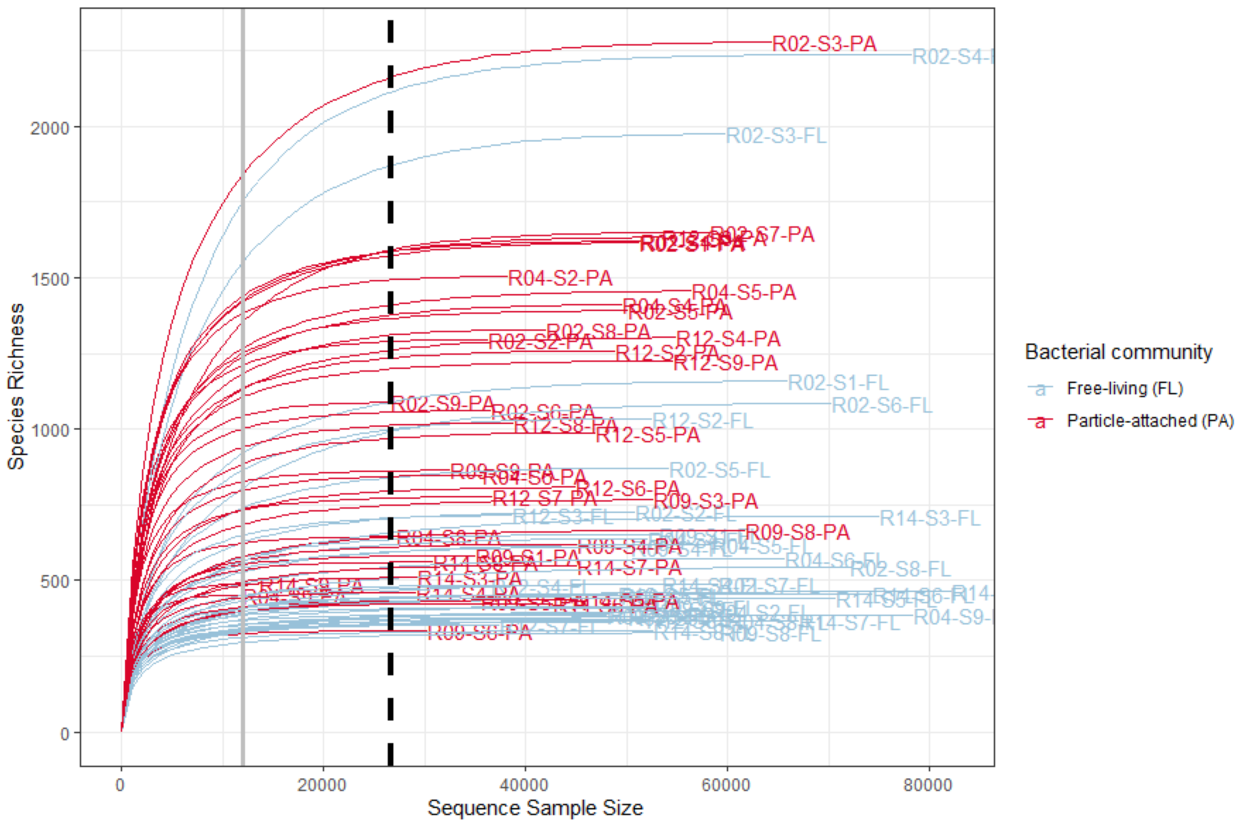

Figure S1. Rarefaction curves of all samples. The grey line represents the minimum sequencing depth while the black dashed line represents the rarefaction depth used, which resulted in two samples loss (PA community in S9 of April 2019 and S9 of July 2020). Samples are named as “sampling date-sampling station-fraction”. R02: February 2019; R04: April 2019; R09: July 2019; R12: November 2019; R14: July 2020; PA: Particle-attached; FL: Free-living.

	Station	Salinity	Temperature (°C)	DIN (µM)	Phosphate (µM)	Silicate (µM)	DOP (µM)	SPM (mg/L)	%POM (%)	Chla (µg/L)	%Chla (%)	%POP (%)
**R02 - February 2019**	S1	0	6.3	456	0.55	155	0.16	5	43	0.29	19	55
	S2	0	6.3	454	0.53	155	0.12	4.9	40	0.30	19	51
	S3	5	6.9	366	0.72	121	0.18	197	15	2.12	14	38
	S4	10.8	7.4	303	0.74	99	0.17	102.9	16	1.59	17	41
	S5	14.7	7.7	258	0.76	84	0.15	40	20	1.09	23	41
	S6	20.2	8.1	181	0.77	62	0.19	44.6	21	0.96	21	41
	S7	26	8.4	111	0.64	39	0.2	19.5	24	0.76	34	42
	S8	29.5	9.0	67	0.57	25	0.13	9.1	30	0.68	42	49
	S9	33.6	10.0	18	0.48	9	0.13	12.4	26	0.75	45	67
**R04 - April 2019**	S2	0	12.7	352	0.28	120	0.25	3.3	37	0.76	28	58
	S4	9.8	12.7	237	0.75	86	0.24	112.3	15	2.97	25	16
	S5	15.6	12.6	181	0.92	68	0.25	58.6	15	2.13	32	38
	S6	20.3	12.5	132	0.64	47	0.29	25.8	18	1.75	39	42
	S8	30.0	12.3	40	0.24	14	0.28	10.8	29	1.70	59	58
	S9	34.1	11.6	4	0.08	2	0.25	1.7	73	2.76	63	67
**R09 - July 2019**	S1	0	23.4	207	0.22	120	0.39	6.9	40	16.1	69	59
	S3	4.4	23.8	198	0.46	104	0.46	42	25	26.1	65	40
	S4	9.9	23.4	136	1.27	83	0.53	74	18	16.7	49	39
	S5	15.3	23.4	108	1.33	63	0.45	40	20	18.6	61	36
	S6	19.6	23.3	72	1.14	47	0.56	24.3	26	18.9	65	45
	S8	29.5	22.5	25	1.19	17	0.49	17.7	22	3.22	52	38
	S9	34.7	18.1	0.5	0.16	2	0.32	13.9	26	1.49	54	56
**R12 - November 2019**	S2	0	8.9	420	0.67	158	0.22	9.7	27	0.44	18	49
	S3	5.5	9.5	330	0.57	122	0.18	55.5	16	0.74	15	49
	S4	9.8	10.0	281	0.64	106	0.2	24.4	17	0.71	23	43
	S5	14.6	10.4	221	0.7	87	0.26	23.4	19	0.54	23	41
	S6	21.1	11.0	147	0.7	61	0.26	19.2	17	0.57	30	41
	S7	24.7	11.3	105	0.64	45	0.24	11.8	19	1.00	49	51
	S8	28.8	10.9	62	0.7	29	0.25	7.4	21	0.93	57	46
	S9	33.3	12.5	18	0.54	10	0.23	4.7	26	0.40	28	
**R14 - July 2020**	S3	4.8	21.4	208	0.66	105	0.39	24.7	25	20.7	57	52
	S4	9.8	20.8	170	0.69	83	0.67	27.8	24	19.0	60	49
	S5	15.6	21.0	121	0.52	58	0.48	13.2	34	14.2	62	54
	S6	20.6	20.5	99	0.52	41	0.48	12.4	31	7.78	54	52
	S7	25.1	20.6	50	0.46	26	0.52	12.6	20	5.65	51	49
	S8	29.5	20.1	25	0.32	11	0.43	8.6	24	3.87	53	46
	S9	34.4	16.7	0.3	0.05	2	0.33	1.8	55	2.01	62	72

Table S1. Physicochemical variables for all samples. DIN: Dissolved inorganic nitrogen; DOP: Dissolved organic phosphorus; SPM: Suspended particulate matter; %POM: Particulate organic matter (in percent of SPM); Chl*a*: Chlorophyll *a*; %Chl*a*: Chl*a* in percent of total pigments; %POP: Particulate organic phosphorus in percent of total particulate phosphorus.

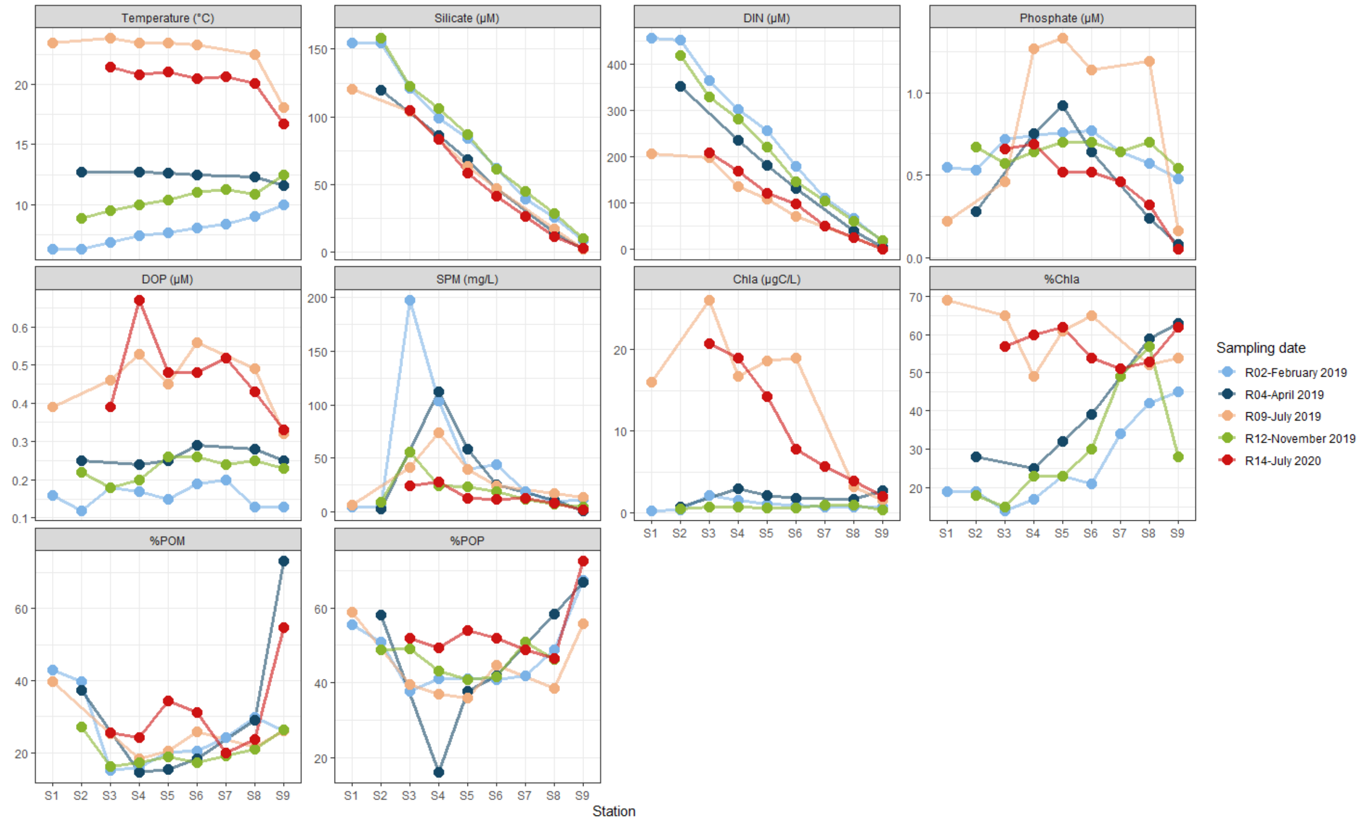

Figure S2. Evolution of the physicochemical variables along the Aulne salinity gradient for all sampling dates.

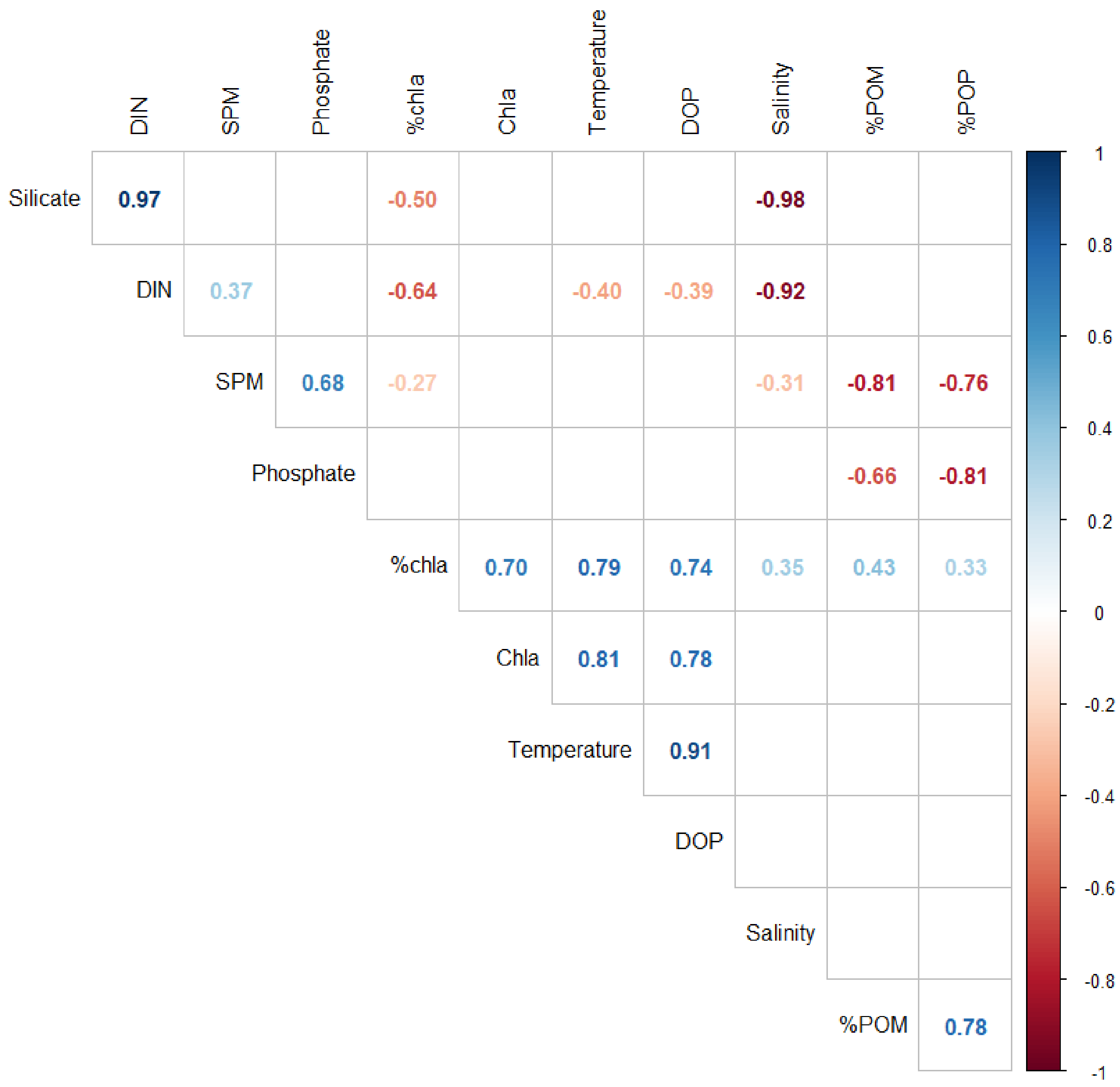

Figure S3. Spearman correlation coefficients of the environmental variables. The variables are grouped according to an “hclust” clustering. Only significant correlations ($p < 0.05$) are displayed.

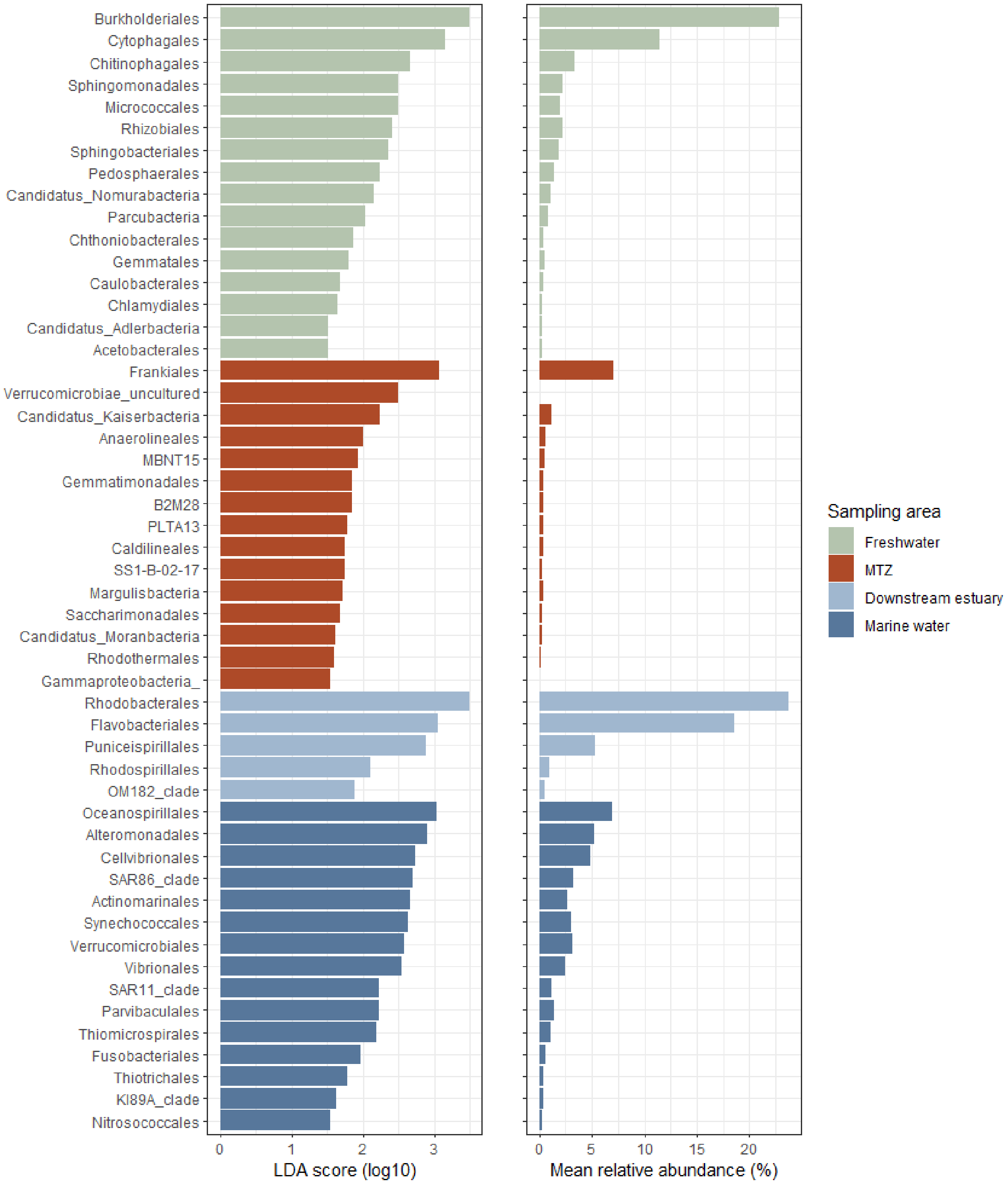

Figure S4. Orders identified by the linear discriminant analysis effect size (LefSe) as most likely to explain the differences between the different areas of the estuary (freshwater, MTZ, downstream estuary, marine water). Left: linear discriminant analysis (LDA) scores; Right: mean abundance in the enriched area.

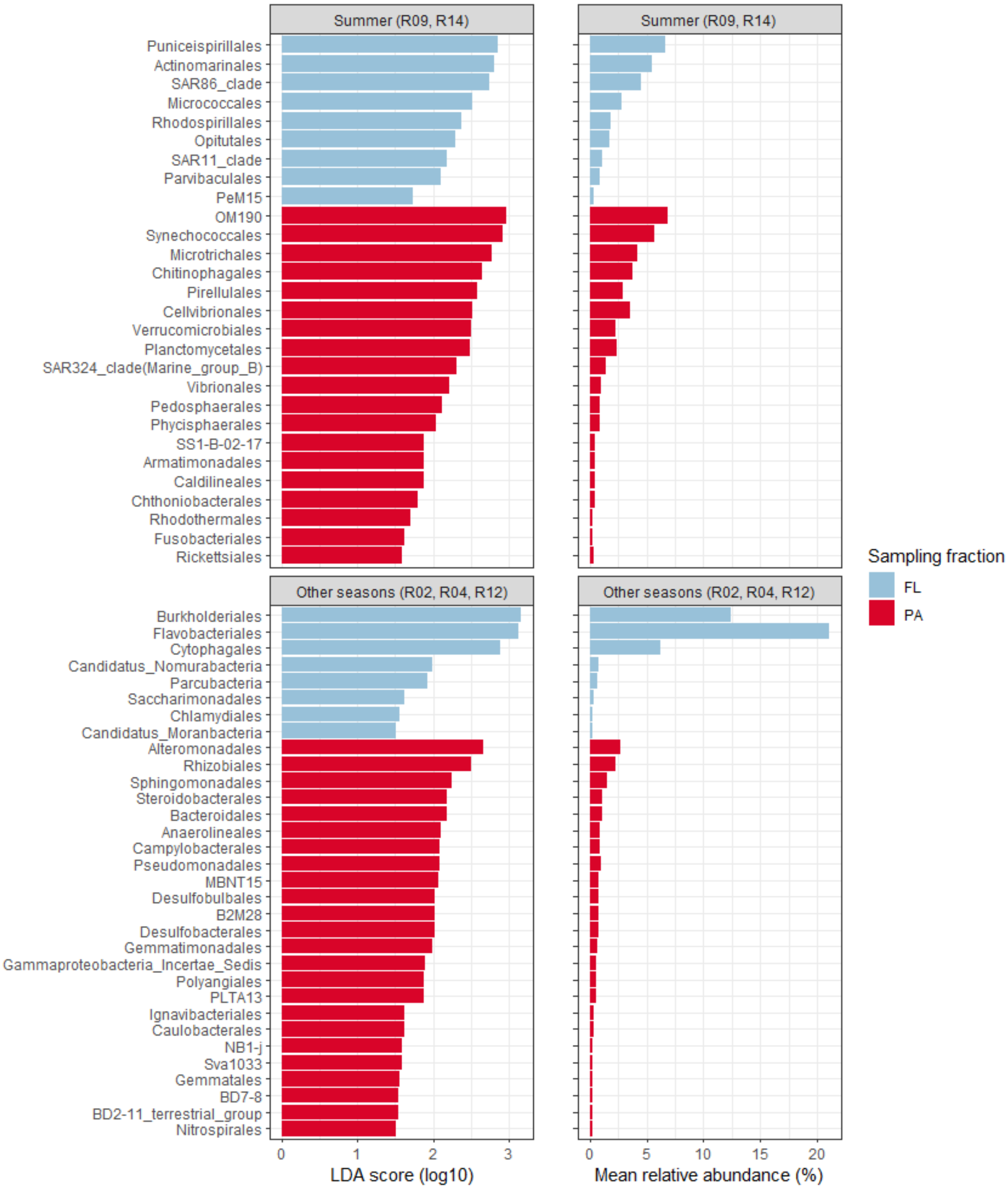

Figure S5. Orders identified by the linear discriminant analysis effect size (LefSe) as most likely to explain the differences between four groups: PA communities in summer, FL communities in summer, PA communities in the other seasons and FL communities in the other seasons. Left: linear discriminant analysis (LDA) scores; Right: mean abundance in the enriched area. Cut-off LDA score: 1.5; cut-off p-value: 0.05.

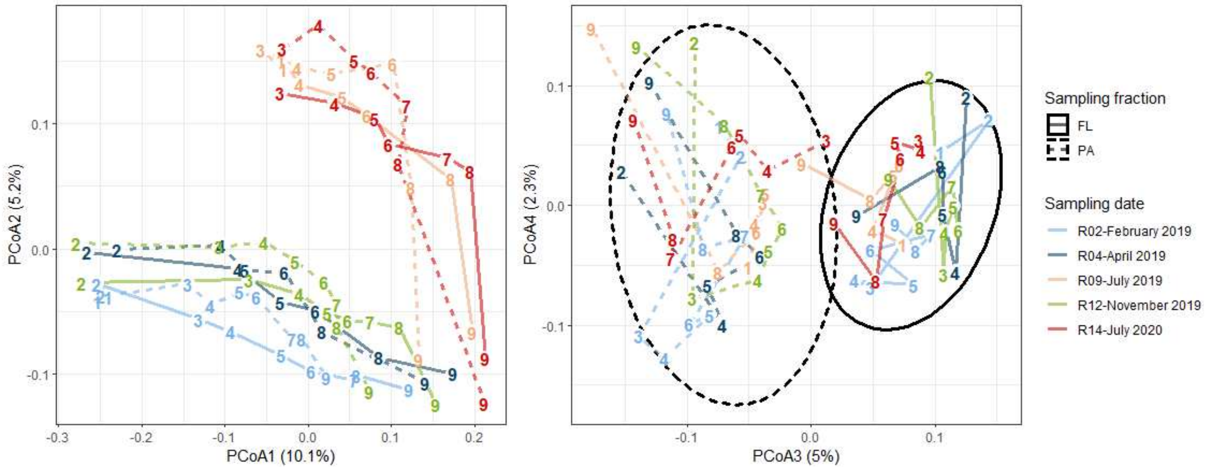

Figure S6. PCoA ordination of all samples based on the abundance-weighted βMNTD (left: axes 1-2; right: axes 3-4). The lines link the different stations within one sampling time in their spatial order (S1 to S9) (solid line: FL communities, dashed line: PA communities). Samples are labeled as stations. PA: Particle-attached; FL: Free-living.

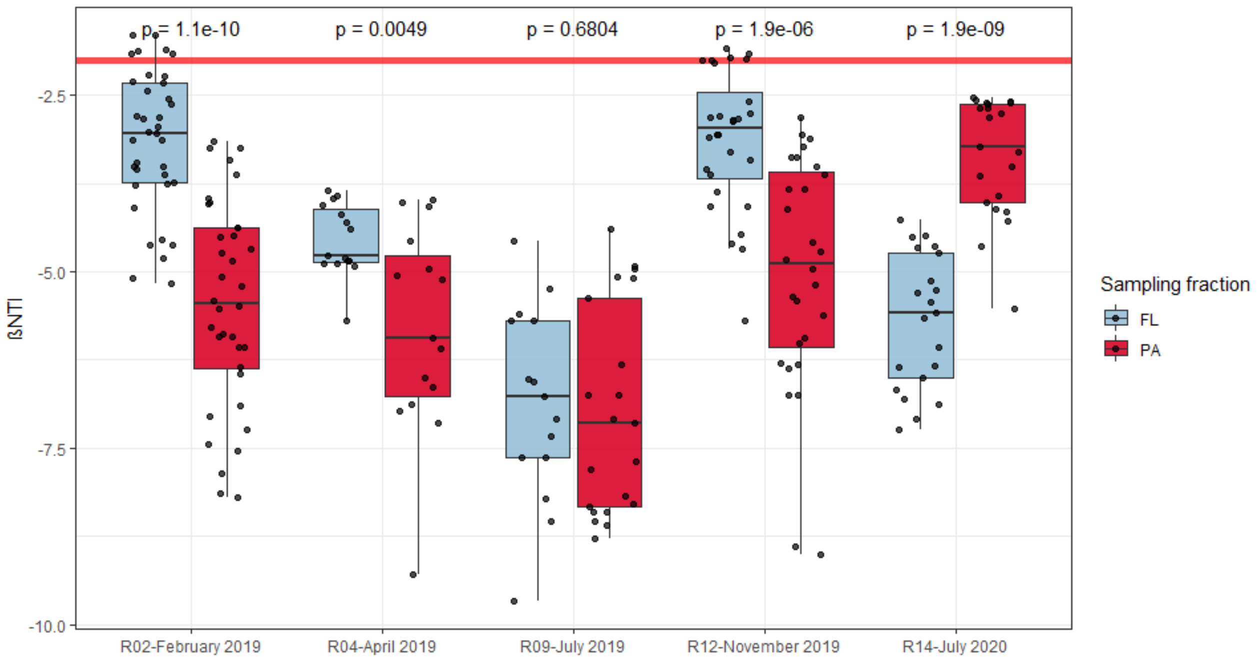

Figure S7. βNTI values for the FL and PA fractions across the five sampling dates. The βNTI < -2 threshold (red line) indicates the predominance of deterministic homogeneous selection. The p-values compare the mean βNTI of the FL and PA fractions for each sampling date (Wilcoxon test).
